# Research Letter: Flooding, displacement, peritraumatic experience and disaster-related PTSD in northern New South Wales – The critical need for quality data to plan mental health support

**DOI:** 10.1177/00048674231203901

**Published:** 2023-10-04

**Authors:** Jo Longman, Kazi Rahman, Veronica Matthews, James Bennett-Levy

**Affiliations:** University Centre for Rural Health, The University of Sydney, Lismore, NSW, Australia

## Introduction

Northern NSW (NNSW) is a ‘hotspot’ for disasters, particularly floods. Floods can have powerful impacts on mental health, including post-traumatic stress disorder (PTSD). Previous disaster research has found that peritraumatic experience – ‘*. . . the emotional and physiological distress experienced during and/or immediately after a traumatic event*’ (Bunnell et al., 2018) – is highly associated with subsequent development of PTSD. In this Research Letter, we present updated findings from a NNSW study in 2017 of the link between peritraumatic experience and PTSD (not previously included in papers published from this study). These data are particularly pertinent as in 2022 there was more extreme flooding in NNSW with numerous reports of peritraumatic experience, which would suggest there is likely to be a huge unmet need for effective PTSD treatment in the coming years.

## Methods

Following major flooding in NNSW in 2017, our team at the University Centre for Rural Health (part of the University of Sydney based in NNSW) undertook a cross-sectional survey on paper and online, measuring the mental health and well-being of the NNSW community 6 months following the 2017 floods. A community-academic partnership enabled a broad recruitment strategy with a focus on recruiting minority groups (e.g. Indigenous, socio-economically disadvantaged) known to be disproportionately affected by disasters (Matthews et al., 2019).

We assessed several exposure measures including inundation of flood water in respondents’ homes and/or businesses, displacement from home and peritraumatic experience at the time of the flood, and several important mental health outcomes known to be of concern following a disaster, including PTSD. The survey estimated probable PTSD among respondents using the Posttraumatic Stress Disorder Checklist - (PCL-6), a validated measure comprising a series of agreement/disagreement statements about recent experiences of intrusive memories, numbing/avoidance and hyper-arousal symptoms that people sometimes have after severe rain and flooding. The diagnostic performance of this measure has proved satisfactory in primary care settings, including for minority populations (sensitivity, 80–92%; specificity, 72–76%) (Spoont et al., 2013).

The study was approved by the University of Sydney Human Research Ethics Committee (reference – 2017/589) and the Aboriginal Health and Medical Research Council Human Research Ethics Committee (reference – 1294/17).

## Results

In total, 2530 respondents participated, all of whom provided their informed consent for inclusion before completing the questionnaire. We reported that people whose homes and/or businesses were inundated were at particularly high risk for probable PTSD (adjusted odds ratio = 13.72; 99% confidence interval [CI] = [4.53, 41.56]) as were those who had been displaced from home for more than 6 months (adjusted odds ratio = 24.43; 99% CI = [7.05, 84.69]) compared with respondents who were not exposed to the flood (Matthews et al., 2019).

Here, we report a new analysis from our 2017 flood study which indicates that peritraumatic experience was highly associated with probable PTSD at 6 months. For example, of 255 respondents in 2017 who thought they may be badly injured or feared for their life during the flood, 115 (45%) reported probable PTSD at 6 months compared with 247 (12%) among the 2071 who did not report a peritraumatic experience (Table 1). The (adjusted) odds of reporting probable PTSD for the 139 respondents who thought they may be badly injured or feared for their life and thought a loved one might be badly injured or their life was in danger, and felt terrified, hopeless or helpless at the time of the flood was 6.5 times greater (95% CI = [4.4, 9.5]) than respondents who did not report these peritraumatic experiences.

**Table 1.** Peritraumatic experience during flood and probable PTSD at 6 months (*n* = 2326).

Peritraumatic experience^a^	Probable PTSD^b^		Odds ratio [95% CI]	
	Without	With	Crude	Adjusted
Thought of being badly injured or feared for life				
No [*N* = 2071]	1824 (88.1%)	247 (11.9%)	Reference	Reference
Yes [*N* = 255]	140 (54.9%)	115 (45.1%)	6.1 [4.6, 8.0]	5.9 [4.3, 7.9]
Thought a loved one might be badly injured, or their life was in danger				
No [*N* = 1866]	1649 (88.4%)	217 (11.6%)	Reference	Reference
Yes [*N* = 460]	315 (68.5%)	145 (31.5%)	3.5 [2.8, 4.5]	3.5 [2.7, 4.5]
Felt terrified, helpless or hopeless				
No [*N* = 1792]	1665 (92.9%)	127 (7.1%)	Reference	Reference
Yes [*N* = 534]	299 (56.0%)	235 (44.0%)	10.3 [8.0, 13.2]	10.3 [7.8, 13.6]
Thought of being badly injured or feared for life, AND thought a loved one might be badly injured, or their life was in danger, AND felt terrified, helpless or hopeless				
No [*N* = 2216]	1925 (86.9%)	291 (13.1%)	Reference	Reference
Yes [*N* = 139]	299 (48.2%)	235 (51.8%)	7.1 [5.0, 10.1]	6.5 [4.4, 9.5]

PTSD: post-traumatic stress disorder; CI: confidence interval.

All are row percentages of not developing and developing probable PTSD within each of the values of the peritraumatic experience variables. Odds ratios are adjusted for age, sex, Aboriginal and Torres Strait Islander status, relationship status, educational qualification, employment status, and income support.

a Peritraumatic experience indicators from the Brief Weather-Related Disaster Trauma Exposure and Impact Screen developed by Helen Berry and colleagues and used in Clemens et al. (2013). Described in detail in Appendix A in Matthews et al. (2020).

b Posttraumatic Stress Disorder Checklist - PCL-6 tool used to measure probable PTSD (Lang and Stein, 2005).

## Discussion

In 2022, NNSW experienced the most devastating floods in Australia’s history in terms of the number of homes affected. In April 2022, State Emergency Services reported that 8108 homes had been inundated and over 4000 houses left uninhabitable. There are no official government estimates of numbers of people displaced, but figures of somewhere between 15,000 and 20,000 people were widely reported.

At 1-year post-flood, around 2000 people are still seeking government-assisted accommodation (housing pods and emergency accommodation). Furthermore, it is thought that at least as many again have moved back into still flood-affected homes or are living with friends or relatives or have moved from the region. It is of great concern that precise numbers of people still displaced or inadequately housed are not known.

In 2022, not only were far more homes inundated and people displaced than in 2017, but the floods were more life-threatening. Again, accurate data are sparse, to our knowledge, there has been no local research which has included similar measures to ours, but we do know that the NSW State Emergency Service undertook 1636 flood rescues (State Government of NSW, 2022). In addition, there were countless stories of rescues from roofs by members of the public in their tinnies and kayaks or climbing to rescue people stranded by landslips. It is now known that five people in NNSW lost their lives in the 2022 floods.

This *Research Letter* aims to ‘sound the warning’, to alert the readership to the likely huge unmet need following a disaster like a flood where home or business inundation, being displaced for a long time and peritraumatic experience was common. The data on peritraumatic experience from 2017 presented here, combined with the previous 2017 inundation and displacement data, indicate that the number of people with PTSD following the severely traumatic 2022 NNSW floods will be in their thousands. However, without better quality data on both exposure and outcomes, it is not possible to predict just how many people have had their mental health compromised. What is required is a robust estimation of the prevalence of PTSD in the flood-affected community. To do this, a suitable sampling strategy (taking the geographic area that was affected and estimating the population at the time of the flood) needs to be designed and implemented as well as a mechanism for reaching households as significant numbers of residents move away from the area following a disaster. This approach will need pilot testing in the local setting. The sampling strategy can then inform the future systematic collection of data as a standard dimension of the formal disaster response. Having these data would provide evidence to underpin lobbying for additional PTSD support in the community, although we acknowledge that the route from such evidence to appropriate mental health support is far from straightforward.

At this stage, it is unclear how the unmet need of, possibly, thousands of people with PTSD in NNSW might best be addressed. The treatment of PTSD is a specialised area of care. The two most evidence-based PTSD treatments are trauma-focused cognitive-behavioural therapy and eye-movement desensitisation reprocessing (Mavranezouli et al., 2020). These are individual-based treatments, requiring highly specialised skills. Currently, there is a shortage of qualified and available mental health support staff and psychologists in NNSW reflected in long waiting lists, and a particular shortage of those with the training and expertise to treat clients with PTSD. Given that social connectedness and feelings of belonging are associated with positive recovery following floods (Matthews et al., 2020), group-based approaches to treatment may be the most pragmatic and cost-effective way forward.

In summary, there is an urgent need for both an assessment of the number of people with flood-related PTSD and for the recruitment and/or training of specialist health professionals to provide evidence-based PTSD treatments for this trauma-exposed population.
